# Epstein-Barr Virus Hepatitis Masquerading as Painless Jaundice

**DOI:** 10.7759/cureus.30333

**Published:** 2022-10-15

**Authors:** Anuoluwapo Adelodun, Amro Abdellatief, Oyedotun Babajide

**Affiliations:** 1 Internal Medicine, Harlem Hospital Center, Columbia University, New York, USA; 2 Internal Medicine, Interfaith Medical Center, New York, USA

**Keywords:** elevated liver associated enzymes, ebv serology, acute viral hepatitis, beta thalassemia minor, ebv- associated hepatitis, painless obstructive jaundice

## Abstract

Epstein-Barr virus (EBV) infection typically presents with pharyngeal symptoms and subclinical transaminitis. We present a case of a 27-year-old woman with no known past medical history who presented with painless jaundice and dark-colored urine for three days. Her review of systems was negative for fever, sore throat, nausea, vomiting, pruritus, or rash. Her last sexual contact was six months ago with a male partner, and she only drank alcohol socially. Family and surgical history were non-significant. Physical examination revealed 3+ bilateral conjunctival icterus without abdominal tenderness or organomegaly. She had elevated transaminases: alanine transaminase (ALT) of 1287U/L and aspartate aminotransferase of (AST) 1057U/L but her alkaline phosphatase (ALP) was only slightly above normal at 109U/L (normal range 35-104U/L), with a direct hyperbilirubinemia - total bilirubin 9.5mg/dl, direct bilirubin 6.8mg/dl; the abdominal ultrasound revealed non-dilated bile ducts. Hepatitis A, B, and C serology was negative, but her EBV serology showed an infection. She had incidental thalassemia minor without splenomegaly or asterixis. She was managed conservatively, and her liver enzymes trended down with supportive management. Although EBV is an uncommon cause of painless jaundice, this diagnosis should be considered, especially when other more common causes of jaundice have been ruled out. A high index of suspicion should be maintained to detect EBV hepatitis as it can easily be diagnosed through serological testing.

## Introduction

Epstein-Barr virus (EBV) infection is common in the United States (US), and worldwide, it has a prevalence of over 90% and is usually asymptomatic in children [1]. In adults, it is spread through saliva by kissing or by sharing food and utensils. Liver involvement, though typical, is usually subclinical. Most adults present with the classic symptoms of sore throat, fever, enlarged lymph nodes, and mild transaminitis [2]. When viral hepatitis is associated with the acute onset of jaundice in a young adult, it is most commonly associated with hepatitis A or B, and a viral prodrome usually precedes both. EBV hepatitis rarely presents with acute jaundice. Our patient seemed to be one of those rare presentations.

## Case presentation

A 27-year-old woman with no known past medical history presented to the emergency department (ED) on account of jaundice and dark-colored urine for three days, associated with pale-colored stools. She is originally from Barbados and frequently travels to the US. Two weeks prior to this presentation, she was in Barbados. One week before her presentation, she returned from a carnival in Miami, Florida. She participated in face painting during her trip. She denied fever, chills, abdominal pain, nausea, vomiting, sick contacts, pruritus, or rash. She had no past surgical history, history of allergies, or family history of jaundice or liver disease. She had never smoked or used illicit drugs and only drank alcohol during social gatherings. Her last sexual contact was six months ago with a male partner. Physical examination revealed no other stigmata of chronic liver disease except 3+ bilateral conjunctival icterus. She had no abdominal tenderness, distention, organomegaly, edema, or asterixis.

**Figure 1:**
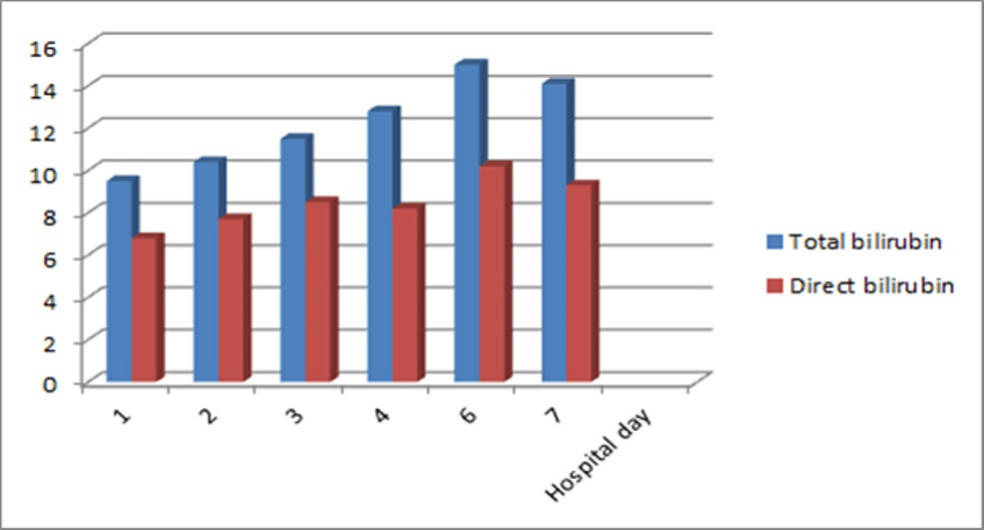
Bilirubin trends during hospitalization

Laboratory evaluation revealed a mixed hepatocellular, cholestatic jaundice. Her transaminases were markedly elevated with alanine transaminase (ALT) of 1287U/L and aspartate aminotransferase (AST) of 1057U/L. Her alkaline phosphatase (ALP), however, was only slightly above normal at 109U/L (normal range 35-104 U/L) with a gamma-glutamyl transferase (GGT) elevated to 220U/L (normal range 5-40 U/L). She had mostly direct hyperbilirubinemia with a total bilirubin of 9.5mg/dl (normal range 0.1-1.2mg/dl) and direct bilirubin of 6.8mg/dl (normal range 0.1-0.3mg/dl). Total and direct bilirubin trended up on the first six days of hospitalization and thereafter began to decline (Figure 1). 

Her international normalized ratio (INR) was slightly prolonged at 1.29. Complete blood count (CBC) revealed a white blood cell (WBC) count of 7.88, platelet count of 339, with normal differentials (neutrophils 48.8%, lymphocytes 37.9%, monocytes 9.5%, eosinophils 2.9% and basophils 0.5%). She was anemic with a hemoglobin of 10.4, mean corpuscular volume (MCV) of 66.2, and red cell distribution width (RDW) of 16.9. Iron studies showed elevated iron 200, normal unsaturated iron binding capacity (UIBC) 136, normal total iron binding capacity (TIBC) 336, elevated transferrin saturation of 59%, and ferritin of 459ug/L. Her lactate dehydrogenase (LDH) was 415U/L (normal range 125-220U/L). Hemoglobin electrophoresis was consistent with beta-thalassemia minor. A direct Coombs test to check for hemolytic anemia came back negative; however, her haptoglobin level was < 20. An abdominal ultrasound showed normal hepatic echogenicity and no dilated intrahepatic or extrahepatic ducts.

The cause of her jaundice was thought to be intrahepatic, so viral studies were sent for hepatitis A, B, and C, which all came back negative. Ceruloplasmin level to screen for Wilson’s disease also came back normal, as did anti-smooth muscle antibodies, anti-liver kidney microsomal antibodies, and antimitochondrial antibodies to rule out autoimmune liver disease. Given the height of her transaminase elevations, the patient was again queried about toxin ingestion. She denied cocaine or other drugs, and her lead level was normal. The endemicity of malaria in Barbados was considered, and testing for the malaria parasite was performed and found to be negative. She was then evaluated for possible acute EBV and cytomegalovirus (CMV) infections. Further laboratory evaluation revealed negative CMV immunoglobulin M (IgM). Her Epstein-Barr virus deoxyribonucleic acid (EBV DNA) was undetectable while her EBV viral capsid antigen (VCA) immunoglobulin G (IgG), EBV early antigen (EA) antibody (Ab) enzyme immunoassay (EIA), Epstein-Barr nuclear antigen (EBNA) IgG EIA, and EBNA IgG came back with high titers (Table 1).

**Table 1:** Results of EBV serology EBV VCA IgM - Epstein-Barr virus viral capsid antibody immunoglobulin M EBV VCA IgG - Epstein-Barr virus viral capsid antibody immunoglobulin G EBV EA Ab EIA - Epstein-Barr virus early antigen antibody enzyme immunoassay EBNA IgG EIA - Epstein-Barr virus nuclear antigen immunoglobulin G enzyme immunoassay

Component	Result	Reference Range	Interpretation
EBV VCA IgM	12.7	<35.9 U/ml	Negative
EBV VCA IgG	>750.0	<17.9 U/ml	Positive
EBV EA Ab EIA	15.8	<8.9 U/ml	Positive
EBNA IgG EIA	114	<17.9 U/ml	Positive

**Figure 2:**
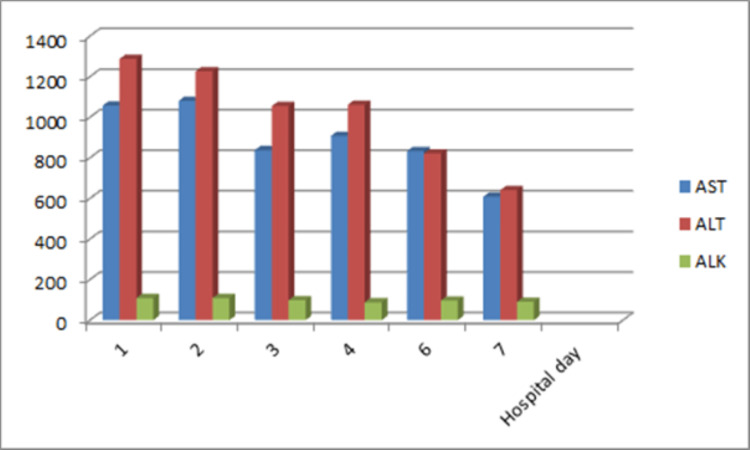
Liver enzyme trends during hospitalization AST - Aspartate aminotransferase ALT - Alanine transaminase ALK- Alkaline phosphatase

Her EBV-VCA IgM came back negative. These findings were consistent with EBV reactivation. Her liver enzymes trended down with supportive management (Figure 2), and she was discharged for follow-up. 

## Discussion

It is estimated that nearly 95% of the adult population has been infected with EBV and that the virus remains latent in B-lymphocytes [3]. The reactivation of EBV infection in the setting of immunosuppression is well known. Although reactivation may also occur without an obvious trigger and with minimal symptoms, it is essential to note that coronavirus disease-2019 (COVID-19) infection may be associated with EBV reactivation in adults. Our patient likely had EBV reactivation. Her presentation varied from acute EBV infection in several respects. First, she was deeply jaundiced but otherwise without the usual symptoms of a viral infection, such as fever, chills, myalgias, arthralgias, nausea, and vomiting. Her jaundice may have been exaggerated by a coincident mild hemolytic anemia due to her underlying thalassemia, as suggested by her low haptoglobin. 

Thalassemia is a group of autosomal recessive conditions that result due to complete or partial absence in the production of globin chains, an important component of hemoglobin. Mutation in the globin chains could lead to instability and intracellular precipitation, resulting in hemolytic anemia by early destruction of red blood cells in the bone marrow [4]. EBV infection in the setting of hereditary hemolytic anemia has been reported to cause jaundice [5]. In addition, her alkaline phosphatase was not as high as has been reported in true cholestatic EBV infections [6]. Jaundice is estimated to occur in only about 6% of acute EBV infections and perhaps 1% of all patients presenting with jaundice of any kind [7-10]. Moreover, the level of her transaminase elevations should be noted. These were both 30 times the upper limit of normal. Transaminase levels are rarely greater than 1000 with EBV hepatitis. Again, this may have been exaggerated due to concurrent hemolytic anemia.

Infectious mononucleosis, characterized by fever, pharyngitis, and lymphadenopathy, is the most common presentation of EBV infection in young adults [11]. Diagnosis is based on a combination of clinical symptoms and EBV-specific serological testing. Primary infection is confirmed by positive immunoglobulin IgM antibodies to EBV VCA. The absence of IgM antibodies in our patient, along with high titers of EBV-VCA IgG, EBNA IgG, and elevated titers of EBV-EA Ab, is very consistent with EBV reactivation [12]. 

The management of EBV hepatitis is mainly supportive, and because it is an uncommon occurrence that mostly resolves spontaneously, few studies are aimed at exploring treatment modalities. Some studies have recommended the use of steroids in complicated cases of EBV infection [13] and in severe cases of EBV hepatitis causing liver failure [14]. The use of antivirals in severe infectious mononucleosis has been studied. A clinical trial of intravenous acyclovir in the treatment of EBV hepatitis has shown no clinical benefit [15]. However, one case study of severe EBV hepatitis treated with valganciclovir ended in the resolution of symptoms [16]. Fortunately, our patient was only treated with supportive measures and made a full recovery.

## Conclusions

Although EBV is an uncommon cause of painless jaundice, some patients may present with an isolated mixed hepatocellular, cholestatic jaundice without other more common symptoms like abdominal pain, fever, chills, myalgias, arthralgias, nausea, and vomiting. In patients that present with isolated jaundice, this diagnosis should be considered, especially when other more common causes of jaundice have been ruled out. It is also important to investigate other factors that may contribute to the pattern of cholestasis, such as autoimmune hemolytic anemias and, in the case of our patient hereditary hematologic disorders. A high index of suspicion should be maintained to detect EBV hepatitis as it can easily be diagnosed through serological testing.
